# Exploring the Potential of Matching‐to‐Sample‐Based Training Protocols to Enhance Language Abilities in Individuals with Language Impairments: A Scoping Review

**DOI:** 10.1111/1460-6984.70260

**Published:** 2026-05-06

**Authors:** J. H. R. Maes, A. R. Scheper, D. Hermans, C. T. W. M. Vissers

**Affiliations:** ^1^ Donders Institute for Brain, Cognition and Behaviour, Centre for Cognition Radboud University Nijmegen The Netherlands; ^2^ Royal Kentalis Utrecht The Netherlands; ^3^ Behavioral Science Institute Radboud University Nijmegen The Netherlands

**Keywords:** developmental language disorder, derived language skills, intervention, matching‐to‐sample, scoping review

## Abstract

**Background:**

Interventions for Developmental Language Disorder (DLD) span explicit and implicit approaches, yet Matching‐to‐Sample (MTS) protocols, a well‐established method for fostering equivalence‐based learning, remain unexamined in this population. The partly incidental and implicit nature of these protocols may align more closely with the way language skills are acquired in everyday contexts.

**Aims:**

To assess their potential for use in individuals with DLD, we conducted a scoping review of MTS‐based language interventions in individuals with language impairments.

**Methods:**

Following the PRISMA‐ScR guidelines and using Web of Science and PsycINFO, sixteen studies (*N*  =  81, primarily children and adolescents) met inclusion criteria, the key requirement being evidence of language difficulties. In most studies, these difficulties co‐occurred with diagnoses of autism spectrum disorder or intellectual disability.

**Main Contribution:**

The review revealed that most interventions targeted foundational receptive and expressive skills and reliably produced untrained (derived) stimulus relations, underscoring the efficacy of implicit learning mechanisms. However, small sample sizes, varied MTS formats, and a dearth of long‐term follow‐up constrain generalizability.

**Conclusions:**

Our findings position MTS as a promising framework for DLD but highlight the need for controlled trials with standardized protocols, larger and DLD‐specific cohorts, and measures of sustained, functional language gains.

**WHAT THIS PAPER ADDS:**

*What is already known on the subject*

- Matching‐to‐sample (MTS) training protocols have been successfully applied to teach a wide range of skills across diverse populations. However, systematic reviews specifically evaluating the potential of MTS‐based language interventions for individuals with language impairments, and for Developmental Language Disorder (DLD) in particular, are lacking.

*What this study adds to existing knowledge*

- This scoping review identified a relatively small number of studies involving individuals with language difficulties, most of whom also presented with co‐occurring neurocognitive conditions. No studies were found that focused specifically on individuals with DLD. Across the available studies, outcomes were generally positive, with evidence not only for gains in explicitly trained language skills but also for the emergence of untrained foundational language skills.

*What are the potential or actual clinical implications of this study?*

- If positive findings are replicated in larger, well‐controlled trials that also address more advanced language abilities, MTS‐based language interventions may represent a promising approach for the sustained improvement of language skills in individuals with DLD.

## 1 Introduction

Developmental Language Disorder (DLD), formerly known as Specific Language Impairment (SLI), is a neurodevelopmental condition characterized by persistent difficulties in language acquisition that cannot be attributed to intellectual disability, hearing impairment, or other neurological disorders (Bishop et al. 2017; Conti‐Ramsden and Durkin 2017). Individuals with DLD struggle with various aspects of language, including phonology, grammar, vocabulary, and complex sentence structure, often leading to long‐term academic and social challenges (Leonard 2014). Despite its high prevalence —affecting approximately 7% of children— DLD remains underdiagnosed and under‐researched compared to other neurodevelopmental disorders (McGregor 2020).

Interventions to stimulate language development in children with DLD typically fall into two broad categories: explicit and implicit approaches. Explicit training involves direct instruction and metalinguistic explanations (e.g., Balthazar et al. 2020; Ebbels 2014). These methods explicitly teach phonological structures, word meanings, and linguistic rules, assuming that structured learning environments help compensate for language difficulties. For example, a common meta‐linguistic technique uses shape and/or color coding to indicate grammatical roles in written sentences, such as distinguishing nouns from verbs, accompanied by explicit instructions. In contrast, implicit learning approaches aim to develop linguistic skills automatically, without explicit instruction (e.g., Baron and Arbel 2022). One example is the “bombardment” technique, in which participants are repeatedly exposed to a target linguistic form through modelling (e.g., Plante et al. 2018). However, in practice, explicit and implicit approaches often overlap (e.g., Baron and Arbel 2022). For example, after implicitly acquiring a grammatical rule, a participant may develop explicit awareness of it (e.g., Bialystok 1986). Conversely, an initially explicitly learned rule may become automatic and applied unconsciously in new contexts (e.g., Ellis 2005).

Some studies suggest that individuals with DLD show impairments in implicit learning under certain conditions (Lammertink et al. 2017; Lum et al. 2014; Obeid et al. 2016), although there is no conclusive evidence of a *general* implicit learning deficit (e.g., West et al. 2021; see also Maes et al. 2025). Implicit approaches reflect the naturalistic way in which typically developing (TD) individuals acquire language —mostly through unconscious absorption of linguistic patterns from exposure (e.g., Hulstijn 2015). This might suggest that implicit approaches are more effective than explicit methods. However, research suggests that for individuals with language difficulties, including those with DLD, interventions that combine implicit and explicit approaches are generally more successful than implicit‐only training (e.g., Finestack 2018; Finestack et al., 2000). Evidence directly comparing explicit‐only versus combined approaches is still lacking. The optimal approach –whether explicit, implicit, or a combined− likely depends on factors such as participant age, cognitive abilities, and the specific language skill being targeted (e.g., Baron and Arbel 2022; Neumann et al. 2024).

### 1.1 Matching‐to‐Sample

One promising intervention approach that integrates both explicit and implicit training elements is the use of Matching‐to‐Sample (MTS) protocols (e.g., Blair and Dorsey 2021; Sidman 1971). In a typical MTS procedure, participants are presented with a sample stimulus, either simultaneously with or followed by two or more comparison stimuli. One comparison is designated as the correct match because it is related to the sample, while the others serve as distractors. After the participant selects a comparison stimulus, accuracy feedback is provided. For example, in the One‐to‐Many (OTM) MTS variant, a trial may present a picture of a dog (sample; A) alongside the written words dog and cat (comparisons). Positive feedback is given for selecting dog (B; training of A‐B relation). In another trial, the same picture of the dog (A) appears with the spoken words “dog” and “cat”, where choosing “dog” (C) is correct, reinforcing the A‐C relation. After baseline training, participants are tested for derived relations without feedback. These post‐training assessments function as a combined equivalence test in the sense described by Sidman and Tailby (1982), because they evaluate whether untrained relations emerge and whether stimuli can function in novel and untrained MTS roles (e.g., former comparisons serving as samples). For example, participants may demonstrate identity matching (A‐A), where they select the dog picture when shown the same image. A symmetry test assesses whether they select A when presented with B (B‐A relation) or C (C‐A relation), thereby reversing the trained direction of responding. A transitivity or combinatorial entailment test evaluates whether they choose C when presented with B (B‐C relation) and vice versa (C‐B relation), relations that were never directly trained and that require recombination of previously established associations. Successful performance across these tests indicates that the stimuli participate in an equivalence class, meaning that A, B, and C are treated as functionally interchangeable despite only some relations having been directly trained.

Although the present example illustrates a OTM arrangement, other training structures are also commonly used in equivalence research. In a many‐to‐one (MTO) arrangement, multiple distinct sample stimuli are trained to relate to a common comparison stimulus, whereas in a linear or serial arrangement, relations are trained sequentially (e.g., A‐B and B‐C), with equivalence demonstrated through the emergence of untrained relations such as A‐C and C‐A. These alternative configurations differ in training structure but rely on the same fundamental MTS logic and assessment of derived relational responding. For schematic representations of these arrangements, readers are referred to Sidman and Tailby (1982) and Green (2001) for a clear description of basic MTS protocols and variants thereof. Importantly, although MTS incorporates explicit elements, such as accuracy feedback, the emergence of untrained relations may largely rely on implicit learning mechanisms.

One MTS variant that has received substantial empirical attention is the Differential Outcomes Procedure (DOP). In a DOP, each correct sample–comparison pairing is followed by a unique and consistently assigned consequence. This contrasts with a Non‐differential Outcomes Procedure (N‐DOP), in which all correct responses produce the same reinforcer or different reinforcers are used without being systematically linked to specific stimulus relations. Across clinical and non‐clinical populations, DOPs typically lead to faster acquisition, higher accuracy, and better retention than N‐DOP arrangements (see McCormack et al. 2019). The advantage of the DOP is commonly explained in two ways. First, the unique reinforcer may become functionally integrated into the stimulus class formed by the sample and comparison, thereby exerting additional discriminative control. Second, consistent stimulus–outcome pairings may generate conditioned expectancies of specific outcomes, which themselves guide responding and facilitate learning.

MTS‐based learning procedures have been used successfully to train relations involving both arbitrary or abstract stimuli, stimuli from different modalities, and various cognitive and other skills. They have also been used to enhance language and cognitive abilities in various populations with communication impairments, including individuals with autism spectrum disorder (ASD) and intellectual disabilities (Raaymakers et al. 2019). However, a preliminary literature search revealed no studies specifically investigating the effectiveness of MTS‐based training for language development in individuals with DLD. This gap motivated the present scoping review, which examined the use of MTS‐based language interventions for improving language skills in other populations with language impairments. The findings may help inform the development of similar interventions for individuals diagnosed with DLD.

Although there are several previously published review(‐like) papers that have explored MTS‐based interventions and that are relevant to the topic of the present review, they do not fully address our research question. For example, Carr and Felce (2000) discussed theoretical issues and empirical findings on the use of MTS‐derived procedures as language interventions for individuals with severe linguistic disabilities, though it was not a systematic review. Jackson et al. (2006) focused primarily on the language skills necessary for MTS‐derived interventions to succeed in individuals with Autism Spectrum Disorder (ASD) and related syndromes, and the review was also not systematic. O'Donnell and Saunders (2003) examined equivalence learning in individuals with language impairments but specifically for those that also had mental disabilities. Additionally, their study lacked methodological details such as specific search terms. Finally, more recently, Shawler et al. (2023) reviewed the effects of procedural parameters in MTS‐based interventions but exclusively focused on individuals with ASD.

### 1.2 The Current Review

Given these limitations, the present systematic scoping review aimed to synthesize existing evidence on MTS‐based interventions for enhancing language skills in populations with language impairments. Specifically, we sought to identify studies that have applied MTS‐based procedures to language training, characterize the methodological features and effectiveness of these interventions, and determine which MTS parameters are most conducive to learning specific target relations. We anticipated identifying a relatively small number of studies with limited sample sizes (including case studies), with a potential publication bias favouring positive outcomes. Additionally, given the relatively straightforward implementation of MTS procedures for foundational language skills, such as vocabulary acquisition, we expected these interventions to primarily target such skills.

## 2 Methods

### 2.1 Selection Criteria

This pre‐registered scoping review (https://osf.io/bvu8z/) was conducted in accordance with the PRISMA‐ScR guidelines (Tricco et al. 2018). Inclusion and exclusion criteria were guided by the Population, Concept, Context (PCC) framework (Peters et al. 2022). The population of interest comprised individuals with language impairments resulting from any underlying cause, including neurodevelopmental disorders and general intellectual disabilities. The review focused on training programs employing variations of the MTS procedure, with or without explicit feedback, aimed at establishing conditional discrimination learning to enhance language‐related abilities. Studies primarily targeting the use of sign language were excluded, as these interventions rely on modality‐specific motor skills and gesture‐based communication systems that differ conceptually and methodologically from spoken or symbolic language training, limiting the comparability of outcomes across studies. Eligible studies included experimental, quasi‐experimental, and single‐case studies conducted in clinical, educational, or laboratory settings. Only empirical, peer‐reviewed studies published in English were considered; no restrictions were placed on the date of publication. Exclusion criteria encompassed animal studies, theoretical or opinion papers, conference proceedings, dissertations, reviews, meta‐analyses, and studies lacking empirical intervention data. In line with the focus on the implicit acquisition of language skills, the review included only those studies that, in addition to explicitly trained relations, assessed the emergence of untrained (i.e., derived) relations among linguistic stimuli. Accordingly, studies that relied solely on simple auditory–visual matching of non‐linguistic or concrete stimuli (e.g., cars, flowers, foods, flags) were excluded, as such tasks do not involve language‐based relational responding or assess the emergence of derived verbal relations. Studies using pronounceable nonsense‐word stimuli alongside real words were eligible, as such stimuli retain phonological and orthographic properties characteristic of language and differ from abstract, non‐verbal stimuli.

### 2.2 Search Strategy

A systematic search was conducted in Web of Science and PsycINFO, accessed via the Ovid interface. The query string used for Web of Science (under “topic”) was: (“matching to sample” OR “conditional discrimination”) AND (language) AND (impairment* OR disabilit* OR delay). For PsycINFO, the query string was: ((“matching to sample” or “conditional discrimination”) and language).mp. and (impairment* or disabilit* or delay).ab, ti. All retrieved articles were stored in EndNote, and duplicates were manually removed prior to screening. The study selection process involved two stages. During the first, the first author screened articles primarily based on titles and abstracts to exclude those that did not meet the source type criteria. A second, in‐depth screening of full texts was conducted by the first author, to assess eligibility based on participant characteristics, training procedures, and study content. The second and third authors independently reviewed half of the 68 reports that had been screened for eligibility. The initial screening by the first author identified 55 reports as excluded and 13 as included. The two independent assessors initially agreed on the inclusion status of 65 reports. For the remaining three reports, the disagreements were as follows: Assessor 1 considered two reports eligible that the first author had excluded, and Assessor 2 questioned the inclusion of one report. All discrepancies were fully resolved through discussion, resulting in a final decision of 55 excluded and 13 included reports.

**FIGURE 1:**
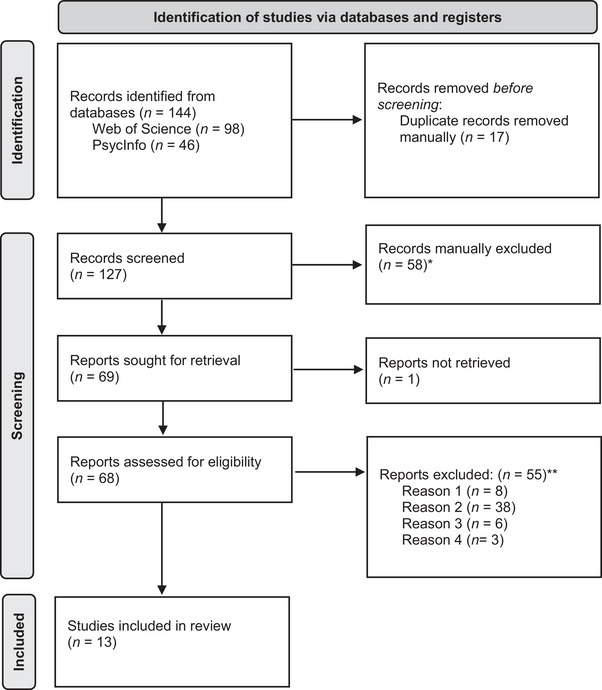
PRISMA 2020 flow diagram for new systematic reviews which included searches of databases and registers only *Reasons for exclusion were: no peer‐reviewed empirical studies (e.g., research proposals, reviews, dissertations, data sets, comments; *n* = 24), no clear training of language function(s) or MTS‐based intervention (*n* = 24), and no human study (*n* = 10). Some studies were excluded for multiple reasons; these studies contributed to only one of the indicted n's, taking only one reason as the primary reason for exclusion. **Reason 1: no (clear) MTS‐based language intervention (e.g., presenting stimulus pairs). Reason 2: no (clear) training of language function and/or testing of derived relations (e.g., studies [mainly] targeting sign language, training of simple auditory‐visual matching involving a very limited set of stimuli [e.g., cars, flowers, foods, flags], use of stimulus sets composed solely of abstract, non‐verbal stimuli as target items, use of MTS only or mainly for assessment purposes, no emergent relations tested, and/or training incorporating non‐linguistic stimuli [including emotional stimuli] as main training target). Note that studies using nonsense‐word stimuli in addition to real words were eligible for inclusion, as such nonsense words retain phonological/orthographic linguistic properties and differ from abstract, non‐verbal stimuli. Reason 3: no language difficulties, whether neurodevelopmentally based or arising from other causes. Reason 4: full‐text not in English. Some studies were excluded for more than one of these reasons. These studies contributed to only one of the indicted n's, taking only one reason as the primary reason for exclusion.

In the pre‐registration, it was mentioned that, following the second screening, the reference list of selected articles would be reviewed for additional relevant studies. However, this screening revealed that these studies primarily employed slight methodological variations rather than new insights. Given that this is a scoping review and in the interest of conciseness, we decided to not include studies from the reference lists in our final selection. The study selection process, including reasons for exclusion, is presented in Figure 1 (PRISMA flowchart).

### 2.3 Data Extraction

Data were extracted from the full texts of the selected articles by the first author, and a random subset (5 articles: 38%) was independently reviewed by the second and third authors. Minor discrepancies emerged in the coding of sample‐characteristic details in two cases, which were resolved after additional verification by the first author. Extracted data were compiled in an Excel spreadsheet, including: meta‐data (author(s), year of publication, country), aim of the study, participant characteristics (diagnosis, sample size, gender, age, and IQ/mental age), the trained language domain(s), intervention features (including MTS variants), the response types and stimuli used, the trained and tested derived relations, and study outcomes. Given the heterogeneity of methodologies, findings were synthesized narratively following the Synthesis Without Meta‐Analysis (SWiM) guidelines (Campbell et al. 2020). Summary tables (Tables 1 and 2) provide an overview of extracted data for each included study.

**TABLE 1:** Overview of primary aim and key demographic details of the samples included in the selected articles.

#	Article	Study	Country	Sample	N	M/F	C Age	IQ or MA	Main aim: evaluation of effectiveness of:
1	Benitez and Domeniconi (2023)	1	Brazil	ID and ASD	7	3m/4f	9‐11	IQ <50‐69	Equivalence‐based teaching in different settings ([special] teachers, parents at home or in school) on reading ability
2	Cariveau et al. (2023)	1	USA	ASD and/or language deficits	4	1m/3f	6‐7	NK	Different types of prompting (delayed, exclusion) on conditional discrimination learning including naming (tacting)
3	Carr et al. (2000)	1	UK	Severe IL	3	2m/1f	15‐21	MA 2:1‐2:3	MTS training for equivalence class formation in individuals with severe intellectual limitations
4	Du et al. (2017)	1	USA	Rubinstein‐Taybi syndrome; ASD	3	2m/1f	4‐5	NK	Auditory matching protocol on echoics to words and advanced listener responses
5	Fisher et al. (2019)	1+2	USA Canada	ASD	4	3m/1f	3‐5	MA <2	Treatment package for training initial auditory‐visual conditional discriminations
6	Lane and Critchfield (1998)	1	USA	Down syndrome	2	2f	12‐14	MA 4:1‐4:2	Identity‐MTS‐based procedure to create vowel and consonant stimulus classes
7	McIlvane et al. (1984)	1	USA	Multiple diagnoses including ASD and brain damage	1	1m	18	NK	Receptive exclusion training/testing, also on naming skill
8a	McIlvane et al. (1992)	1	USA	Severe MR	8	5m/3f	12‐22	MA 2:1‐5:6	Receptive exclusion training/testing, also on naming skill
8b	McIlvane et al. (1992)	2	USA	Severe MR	9	9f	40‐78	MA 3:2‐5:11	Receptive exclusion training/testing, also on naming skill
8c	McIlvane et al. (1992)	3	USA	Severe MR/language deficits	6 from Study 2	6f	NK	NK	Receptive exclusion training/testing, also on naming skill
8d	McIlvane et al. (1992)	4	USA	Severe MR/language deficits	3	2m/1f	41‐71	MA 3:0‐5:6	Receptive exclusion training /testing with blank comparison, also on naming skill
9	Rosales and Rehfeldt (2007)	1	USA	Severe MR/Down syndrome	2	1m/1f	34‐58	IQ 36/MA 3:11 IQ 24/MA 1:8	MTS training on establishing manding skills necessary to complete a chained task
10	Speckman‐Collins et al. (2007)	1	USA	ASD with or without cerebral palsy	2	2f	3:5‐4:8	MA 0:11‐1:3 and 1:9‐1:11	MTS on emergence of naming
11	Stromer and Mackay (1992)	1	USA	Academic deficits	3	3m	9:7‐13:11	IQ 67–85	MTS training with complex spoken samples and different sets of stimuli on equivalence class formation and spelling
12	Wilkinson et al. (2009)	1	USA	ID: ASD, MR, and/or PDD or FAS	10	6m/4f	8.2‐19:0	IQ 36–72 (different instruments); not testable for 3 pp	Receptive exclusion training on equivalence class formation
13	Zaine et al. (2014)	1	Brazil	Mild to moderate MD	14 (7 Exp/7 Ctrl)	5m/9f	9‐15	NK	Intervention package including simple and conditional training on reading

*Note*: ASD = Autism Spectrum Disorder; C Age = calendar age in years and months (e.g., 3:5 = 3 years and 5 months); Ctrl = control condition; Exp = experimental condition; FAS = fetal alcohol syndrome; f/m = female/male; ID = intellectual disability; IL = intellectual limitations; IQ = intelligence quotient, as assessed with the specific intelligence test used in the study; MA = mental age in years or, if explicitly indicated, in months; MR = mental retardation; MTS = matching to sample; NK = not known; PDD = pervasive developmental delay.

**TABLE 2:** Key data from the selected studies.

#	Language domain(s) trained and/or involved	Research design and Intervention/MTS variant(s)	Nature of trained and derived response(s)	Nature of stimuli or responses used	(pre)Trained or pre‐existing relations	Tested derived relations	O
1	Receptive (listening, reading) & expressive (writing, naming)	P‐P‐F+multiple baseline+TtC; mixed OTM, MTO, IM; positive (praise and tokens) and negative (verbal) feedback with trial repetition; different educational agents	Selecting stimuli; naming/reading	Dictated words (A), figures (B), printed words (C), dictated syllables (D), printed syllables (E), naming of words (F), naming of syllables (G), writing with syllables (H)	A‐B; A‐C; B‐B; B‐F; B‐G; B‐H; C‐C; C‐H; D‐E	B‐C (trans); C‐B (trans); C‐F (trans + gen [new words]); E‐G (gen)	+
2	Receptive (listening) and expressive (tacting)	P‐P+AATD+TtC; OTO; positive feedback + correction by delayed prompting of comparison (treatment 1) or prompting by exclusion (treatment 2)	Selecting stimuli; naming	Auditory stimuli (questions) (A), visual stimuli (B)	A‐B	B‐naming (gen)	±
3	Receptive (listening, ‘reading’ letter)	TtC with probe trials; mixed IM, MTO with gradually introduction of blank comparison; partial sample‐stimulus control shaping; partial reinforcement during training and testing	Selecting stimuli	Dictated words (A); line drawings (B), printed letters (C), abstract picture‐like stimuli (D)	B‐B; A‐B; C‐B; D‐B	B‐C (sym); B‐D (sym); A‐C (equiv); A‐D (equiv); C‐D (equiv); D‐C (equiv)	+
4	Receptive (listening) and expressive (echoing)	P‐P+TtC+multiple probes; mixed IM, OTM; positive feedback (auditory stimuli, praise, reinforcers) + verbal encouragement and partial physical prompt after incorrect responses	Selecting stimuli; vocal/echoic responses; responding to vocal instructions/ directions	Pictures + corresponding sounds (A), auditory stimuli (words, phrases) (B), comparison words and phrases sharing only part of the sample stimuli (C), vocal directions with visual distractor (incompatible act) (D)	A‐A; B‐B; B‐C	B‐echoic response (gen); D‐following direction (gen)	+
5	Receptive (listening) & expressive (echoing, tacting)	Concurrent multiple baseline design+maintenance tests+TtC; OTO + DOR; positive feedback (praise, preferred item) + error correction (repeating trials, progressive delay identity‐match prompting: picture of correct comparison)	Selecting stimuli; vocal responding (echoing and naming)	Auditory stimuli (single words) (A), pictures of objects (B)	A‐B	B‐tacting (gen) (for 2 pp)	+
6	Receptive (listening, reading) & expressive (naming in terms of verbal categorization)	P‐P‐F+TtC; IM with 1‐, 2‐, or 3‐element (compound) samples + serial presentation of samples and comparisons; positive feedback (icon, auditory) + negative feedback (icon, auditory)	Selecting stimuli; vocal responses (spoken words)	Printed double letters (Ad), printed single letters (As), spoken words “vowel” or “consonant” (B), printed single letter from the two letters from original Ad+B compound sample belonging to same stimulus class (C), printed 4‐letter words (E)	Ad+B‐C	As‐C; B‐C; correct vocalization to letters (e.g., ‘’vowel’’ to ‘’O’’); identification of letters in E by responding with B (generalizations indicative of larger stimulus class formation)	+
7	Receptive (listening) & expressive (naming)	TtC; OTO with follow‐up; positive feedback (allowing to eat selected food item) + exclusion trials	Selecting and eating stimuli; naming	Dictated words or non‐words (A), real food item (B)	A‐B (also with exclusion trials)	A‐B (exclusion trials); B‐naming (gen)	+
8a	Receptive (listening) & expressive (naming)	TtC; OTO; positive feedback (visual, auditory, token, food item)	Selecting stimuli; naming	Dictated words or non‐words (A); line drawings (B)	A‐B (also with exclusion trials)	A‐B (exclusion trials); B‐naming (gen)	+
8b	Receptive (listening) & expressive (naming)	TtC; OTO; partial positive feedback (praise, penny)	Selecting stimuli; naming	Dictated words or non‐words (A); line drawings (B)	A‐B (also with exclusion trials)	A‐B (exclusion trials); B‐naming (gen)	+
8c	Receptive (listening) & expressive (naming)	TtC; OTO; partial positive feedback (praise, penny)	Selecting stimuli; naming	Dictated words or non‐words (A); line drawings (B)	A‐B (also with exclusion trials with alternating procedure)	A‐B (exclusion) trials; B‐naming (gen)	±
8d	Receptive (listening)	TtC; OTO; partial follow‐up; partial positive feedback (token, penny) + partial trial repetition after incorrect	Selecting stimuli	Dictated words or non‐words (A); line drawings (B)	A‐B with blank comparison	A‐B exclusion trials, also with blank comparison (gen)	+
9	Receptive (listening) & expressive (manding)	P‐P‐F+TtC; OTM; follow‐up; positive feedback (praise, possibility to engage in the completed activity) + trial repetition and corrective feedback in case of incorrect response; gestural prompts in case of excessive errors	Selecting stimuli	Dictated names of items (A); pictures (B); printed words (C)	A‐B; A‐C; manding with B to get object to (partially) complete a chained task	B‐A (sym); C‐A (sym); B‐C (trans); C‐B (trans); manding with C to get object to (partially) complete a chained task (gen)	+
10	Receptive (listening) & expressive (echoing, tacting, naming)	Multiple probe design with pretest+TtC; IM; no info about feedback during training	Selecting stimuli (pushing button, pointing); vocal responding (echoing and naming)	Environmental sounds (A); words (B); pictures (C); pictures with corresponding verbal label (C')	A‐A; B‐B; C'‐C	C'‐pointing to C (type of IM); C'‐echoing C (gen); C‐tacting C (gen)	±
11	Receptive (listening) & expressive (spelling/‘writing’)	Multiple baseline design+TtC; mixed IM, OTM, MTO; positive feedback (auditory, visual)	Selecting stimuli	Pictures (A); printed words (B); spoken words (C), selecting letters (D)	A‐A; B‐B; A‐B; B‐A; B‐D; A‐D; A+B‐A; A+B‐D (repeated for different sets); C‐A (for one set)	A‐D (D: repeated for different sets, equiv); A‐B (B: repeated for different sets; equiv); B‐A (B: repeated for different sets; equiv); B‐B (from different sets; equiv); C‐A (C from sets other than during training; equiv); C‐D (equiv); C‐B (C and B: from different sets; equiv)	+
12	Receptive (listening, ‘reading’ of symbols)	TtC; linear; partial positive feedback (auditory, token, edibles, money)	Selecting stimuli (touching); naming	Spoken labels (real or nonsense words) (A); Photos (B); line drawings/symbols (C)	baseline with familiar stimuli: A‐B; B‐C; with novel/nonsense stimuli (exclusion training): A‐B; B‐C	With nonsense stimuli: A‐C (trans); C‐B (sym)	+
13	Receptive (listening, ‘reading’) & expressive (naming [pictures, words, syllables], ‘writing’ with syllables)	P‐P+TtC; mixed IM, OTM; partial positive feedback (praise, edibles: different for different classes of stimuli) + partial negative verbal feedback and trial repetition in case of incorrect response	Selecting stimuli; naming	Dictated words (A); pictures (B); printed words (C); naming pictures (D); naming syllables (E); constructing words with syllables (F); sound of animals and objects (H)	A‐B; B‐B; B‐D; C‐C; C‐F; (relations also tested after training); B+/C+ (simple discrimination); H‐B; H‐C	C‐B; B‐C; C‐D (all gen)	+

*Note*: AATD = Adapted Alternating Treatments design; DOR = differential observing response; equiv = equivalence; gen = generalization; IM = identity matching; MTO = many‐to‐one; O = outcome; OTM = one‐to‐many; OTO = one‐to‐one; P‐P = pre‐post design; P‐P‐F = pre‐post‐follow‐up design; sym = symmetry; trans = transitivity; TtC = training‐to‐criterion design; + = predominantly positive; ± = somewhat mixed results.

## 3 Results

The systematic search for studies identified a total of 144 records from the two databases: Web of Science (98 records) and PsycInfo (46 records). After removing 17 duplicate records, 127 records remained for screening. During the screening process, 58 records were excluded for various reasons, including not being peer‐reviewed empirical studies, not involving the training of language functions or MTS‐based interventions, or not being human studies. Following this, 69 reports were sought for retrieval; however, one could not be retrieved. This left 68 reports for full‐text eligibility assessment. Of these, 55 reports were excluded for various reasons, including a lack of a clear MTS‐based language intervention, absence of a language function training, no testing of derived relations, no involvement of individuals with a neurodevelopmental or communication disorder, and no availability of the full text in English. Ultimately, 13 articles, one of which reported the results of four studies, for a total of 16 studies, met the inclusion criteria and were included in the final review.

### 3.1 Participant Characteristics and Main Aim (Table 1)

The majority of the studies were conducted in Canada and the USA (13 studies), followed by Brazil (2 studies) and the UK (1 study). Across the 16 included studies, there were a total of 81 participants (34 boys/men and 47 girls/women). Ages ranged from 3 to 78 years, with most studies (11) focusing on children, adolescents, or young adults aged 21 years or younger. All participants demonstrated language impairments. In most studies, these impairments were formally assessed using one or more validated instruments, most commonly the Peabody Picture Vocabulary Test (PPVT), but also a range of other instruments, including Gardner Expressive One‐Word Picture Vocabulary Test (EOWPVT), the Verbal Behaviour Development Assessment (VBDA), the Inventory for Client and Agency Planning (ICAP), and the Preschool Language Scale (PLS). In Studies 7, 8b, 8c, and 13, language impairments were identified through observation rather than standardized testing. With the exception of Studies 2 (for three participants) and 11, participants’ language impairments were linked to a clinical diagnosis, including autism spectrum disorder (ASD), (severe) intellectual disability, Down syndrome, or Rubinstein‐Taybi syndrome. When reported, IQ or mental age levels were generally low, with several participants exhibiting mental ages significantly below their chronological age. In most studies, the specific individuals conducting the training were not detailed, often referred to as the “experimenter”, “instructor”, or “trainer”. In some cases, the training was carried out by a therapist, (school) teacher, and/or parent (data not shown in the table). The main aim of most studies concerned an evaluation of the effectiveness of some variant of MTS‐based learning protocol or program (see below for further details).

### 3.2 Intervention Characteristics (Table 2)

#### 3.2.1 Involved Language Domains

Most studies involved aspects of both receptive and expressive language skills; only three studies exclusively focused on receptive skills (Studies 3, 8d, 12). Regarding receptive skills, 11 studies exclusively involved listening skills (Studies 2, 4, 5, 7, 8a‐d, 9, 10, 11) and five studies involved both listening and reading (Studies 1, 3, 6, 12, 13). Listening skills included the presentation of dictated real and/or nonsense words, syllables, questions, or directions as samples and/or comparisons. Reading skills involved the silent reading or interpretation of printed real and/or nonsense words, letters, syllables, or symbols (lexigrams).

Among the studies assessing expressive skills, one focused exclusively on writing skills (Study 11), ten targeted vocal expression (Studies 2, 4, 5, 6, 7, 8a, 8b, 8c, 9, 10), and two included both writing and vocal expression (Studies 1, 13). Writing skills encompassed “spelling” and “writing” by selecting individual letters, syllables, or words. Vocal expression skills included echoing, tacting, naming (or labelling), reading aloud, categorization, and manding. Echoing refers to the repetition of words or sounds produced by another speaker. Tacting typically involves providing a verbal label (e.g., saying “dog”) in response to a non‐verbal stimulus (e.g., seeing a picture of a dog). The term naming is often used to describe both the act of labelling and the demonstrated understanding of the concept, in response to either verbal or non‐verbal stimuli (e.g., saying “dog” when shown a picture of a dog or the written word “dog”, with an implicit grasp of the concept of a dog). While the terms tacting and naming have distinct theoretical meanings, they are frequently used interchangeably in both practice and the reviewed literature. Finally, manding refers to requesting a specific item or action, typically when the speaker is motivated to obtain it. Ten studies examined tacting or naming of linguistic units (e.g., words, syllables) or visual stimuli, including pictures, line drawings, and food items (Studies 1, 2, 5, 6, 7, 8a, 8b, 8c, 10, 13). In addition, two studies investigated echoing of words and phrases (Study 4, 10), one focused on categorization, specifically identifying whether a letter was a vowel or consonant (Study 6), and one explored manding, in which participants used pictures or printed words to complete a chained task (Study 9).

#### 3.2.2 General Research Design

All studies employed some form of a trials‐to‐criterion training procedure, typically requiring at least some percentage of correct (unprompted) responses before a participant was considered to have mastered a particular phase and/or advanced to the next stage of training or testing. This procedure aligns with an incidental or implicit training procedure because the participants were not explicitly instructed to reach a specific performance level and how they could reach this. Six studies (Studies 1, 2, 4, 6, 9, 13) used a more‐or‐less clearly defined pre‐training/post‐training assessment design. Of these, three (Studies 1, 6, 9) also included a follow‐up assessment. Several of the remaining studies—including some that also used a pre‐post design—employed either a multiple baseline or multiple probe design. In a multiple baseline design, baseline performance is measured across different conditions (e.g., stimulus sets), and the intervention is introduced at different times across conditions. This allows researchers to attribute observed changes to the intervention itself rather than to external factors. A multiple probe design is similar, but instead of continuous measurement, it uses intermittent assessments (probes) taken at selected time points, which helps avoid potential learning during the baseline phase. Studies 7, 8a–d, and 12 did not use any of these designs. Instead, they focused on exclusion training as the primary instructional method, gradually introducing basic stimulus relations as a foundation (see below for details).

The concrete operant responses required from participants included selecting comparison stimuli either by touching a screen or pressing buttons in computerized tasks, or by pointing or touching in paper‐and‐pencil tasks. Additionally, as shown in Table 2, 12 studies also required vocal responses, primarily naming. Trial‐based reinforcement was used to strengthen correct responses in nearly all included studies, typically on a partial reinforcement schedule. Reinforcers included praise, tokens, money, preferred items (including edibles), access to favoured activities, and/or positive auditory or visual cues. All studies employed such reinforcement following correct responses, with the exception of Study 1, which did not report any feedback procedures. In four studies (Studies 6, 8b, 9, 11), generic feedback was also provided. This feedback was not contingent on each individual response but was delivered intermittently, either after a block of trials or during probe trials (for the latter regardless of response accuracy). It typically involved praise, money, or tokens that could be exchanged for primary reinforcers. Corrective feedback following incorrect responses (excluding prompts) was reported in six studies (Studies 1, 5, 6, 8d, 9, 13). This feedback included verbal feedback (e.g., “no, this is not right”), repetition of the trial, or the presentation of negative icons and sounds to signal an incorrect response.

#### 3.2.3 Training Formats

The training formats used across the included studies varied in structure. Seven studies employed an exclusively one‐to‐one (OTO) format (Studies 2, 5, 7, 8a, 8b, 8c, 8d), while two studies used identity matching (IM; Studies 6, 10). One study each applied a one‐to‐many (OTM) training (Study 9) and linear training alone (Study 12). Four studies incorporated a mixture of IM, OTM, and/or many‐to‐one (MTO) formats (Studies 1, 3, 4, 11).

In IM, participants are required to match a sample stimulus to an identical comparison stimulus (e.g., matching a picture of a dog to the same picture: A‐A). A OTO format refers to a procedure in which a single sample stimulus (or class of stimuli) is consistently matched to one specific comparison stimulus (schematically: A‐B). For example, a spoken word such as “dog” may be matched to a line drawing of a dog. In a OTM format, a single sample stimulus is associated with multiple comparison stimuli (A‐B, A‐C). For example, the spoken word “spoon” might be matched to a picture of a spoon on one trial and to the printed word “spoon” on another. The MTO format involves multiple distinct sample stimuli being matched to a single comparison stimulus (e.g., A‐C, B‐C). An example would be matching both the printed and spoken word “dog” to the same line drawing of a dog. Finally, linear training entails a sequence of associations. For example, the spoken word “dog” may first be matched to a photo of a dog (A‐B), and then in subsequent training, the photo becomes a sample to be matched to a line drawing of a dog (B‐C).

#### 3.2.4 Prompting

A variety of prompting strategies were employed across the included studies. In six studies (Studies 2, 4, 5, 9, 12, 13), prompts were directed at the comparison stimuli and were used during the initial phases of training or following incorrect responses in later stages. These prompts included: 1) eliminative prompting, where incorrect comparison stimuli were removed, leaving only the correct one, 2) prompting by exclusion, in which incorrect comparisons were replaced with known stimuli associated with other sample stimuli, 3) highlighting of the sample and correct comparison, 4) gestural prompting, involving physically pointing to or otherwise indicating the correct comparison stimulus, and 5) identity‐matching prompting, involving the presentation of a stimulus identical to the correct comparison (e.g., holding up a matching picture) to guide the participant's selection. Prompting by exclusion refers to a teaching strategy where a participant selects a correct comparison stimulus by *excluding* those that are already associated with known stimuli. For example, a participant has first learned to match the spoken words “dog” and “cat” to their respective pictures. Later, the participant is presented with a new spoken word, “bird”, which has to be matched to one of the three pictures, a picture of a dog, cat, and bird. Even without knowing what “bird” means, the participant might exclude the known matches (dog, cat) and choose the unfamiliar picture (bird).

A prompt directed at the sample was used in Study 5, and consisted of the requirement to echo the sample stimulus before selecting a comparison, a technique known as a differential observing response (DOR). This response could itself be prompted through vocal instructions (e.g., “say dog”) when necessary. Two studies (Studies 4, 10) (also) included general instructional prompts, such as explaining the training procedure or providing feedback like “pay attention” or “try again” following incorrect responses. The remaining studies (Studies 1, 3, 6, 7, 8a, 8b, 8c, 8d, 10, 11) did not report the use of a prompting strategies.

#### 3.2.5 Derived Relations

The reviewed studies varied in the type of derived relations they assessed. Symmetry was examined in three studies (Studies 3, 9, 12). For example, in Study 3, participants were first taught to match line drawings of everyday items to the first letter of the item's name (i.e., A‐B). They were then tested on whether they could select the correct line drawing when presented with the corresponding letter (i.e., B‐A), thus representing symmetry.

Transitivity was assessed in three studies (Studies 1, 9, 12). This typically involved participants learning to match both pictures and printed words to dictated words (A‐B and A‐C). They were then tested on their ability to match printed words to the corresponding pictures (B‐C) and vice versa (C‐B), indicating the emergence of transitive relations.

Equivalence, in the common sense of the term (i.e., the emergence of both symmetry and transitivity), was examined in one study (Study 3). Participants in this study learned to match line drawings to dictated words (A‐B) and to printed letters (C‐B). During testing, they were asked to match the printed letters to the corresponding dictated words (A‐C). This A‐C relation reflects transitivity via the shared B node, although one of the trained relations (C‐B was learned in the reverse direction. The derivation of a B‐C relation from a C‐B history further implies a symmetrical relation.

A total of 13 studies (Studies 1, 2, 4, 5, 6, 7, 8a, 8b, 8c, 9, 10, 11, 13) explored generalized or generalized derived relations in some form. For example, Study 1 assessed generalization of derived associations between printed words and their spoken forms by presenting novel words. This tested whether participants could spontaneously name new words consisting of new combinations of syllables previously taught and of entirely new words. Studies 2, 5, 7, and 8a, 8b, and 8c involved training participants to match visual or real items to auditory stimuli (A‐B), followed by tests in which they were asked to tact or name the item (B‐naming response). Study 10 similarly assessed echoic and tacting responses following identity matching with visual/auditory compound sample stimuli and pointing as response. In Study 4, participants were tested for spontaneously producing echoic responses and following verbal directions after training to match pictorial/sound compound stimuli or spoken words and phrases to identical stimuli (identity matching). Study 6 involved compound sample stimuli consisting of printed letters and a spoken word (“vowel” or “consonant”). The correct comparison matched only one letter in the compound. Subsequent test trials demonstrated participants’ spontaneous verbal categorization of letters as vowels or consonants, indicating the formation of broader stimulus classes. Study 9 assessed spontaneous manding responses using printed words, following training to associate dictated item names with pictures and printed words, and manding with pictures to select concrete objects. In Study 11, participants were trained on associations among pictures, printed words, spoken words, and letter selection, also using picture/word compound samples and often using different sets. Thereafter, they were tested for almost all possible relations, including those involving members across the different sets, providing evidence for the formation of expanded stimulus equivalence classes. Finally, in Study 13, participants were tested on matching pictures to printed words (C‐B) and vice versa (B‐C), as well as reading printed words aloud (C‐D), after training on picture‐dictated word associations (A‐B) and picture naming responses (B‐D).

### 3.3 Study Outcomes Related to Derived Relations

In ten studies, involving 69 participants (all studies except Studies 2, 8c, and 10, which included 12 participants), the majority of the participants responded correctly on more than 75% of the trials for most of the investigated derived relations. In Studies 2, 8c, 9 (only at follow‐up for manding trials), and 10 (total number of participants: 14) the majority of participants reached less than 75% correct responses for most of the examined derived relations. But in all cases, post‐training performance was better than pre‐training performance.

## 4 Discussion

In this scoping review, we synthesized evidence from 13 articles (16 studies; 81 participants) investigating MTS‐based protocols aimed at enhancing language abilities in individuals with language impairments, mostly as a results of a neurodevelopmental condition. Participants ranged from 3 to 78 years of age and represented diverse diagnostic groups (e.g., autism spectrum disorder, Down syndrome, intellectual disability), but none specifically targeted developmental language disorder (DLD). Interventions primarily addressed basic receptive and expressive skills, most often vocabulary acquisition via tacting/naming, and employed various MTS formats, including one‐to‐one (OTO), one‐to‐many (OTM), many‐to‐one (MTO), and identity matching (IM). Across studies assessing derived relations (symmetry, transitivity, equivalence), the majority of participants demonstrated high post‐training accuracy, indicating robust stimulus‐class formation and/or generalization to untrained language tasks. Reinforcement schemes typically combined praise, tokens, and, in some cases edibles.

### 4.1 Methodological Considerations

The considerable heterogeneity in participant characteristics, MTS formats, prompting strategies, feedback types, targeted language domains, and assessed derived relations, together with consistently positive but small‐sample outcomes, precludes identification of specific combinations of MTS parameters, language domains, etc., that optimize language gains. This conclusion aligns closely with that of Shawler et al. (2023), who sought to identify optimal MTS procedural parameters for teaching novel stimulus classes to individuals with ASD but were hindered by the wide variability in parameters. Moreover, the uniform reporting of successful outcomes of the studies included in our review raises concerns about publication bias, as null or negative findings may be underrepresented (see also, Tincani and Travers 2019).

Despite the wide range of MTS formats employed in the reviewed studies, one variant, with demonstrated effectiveness in other research, was notably absent: the DOP. As outlined in the introduction, in this procedure each correct sample–comparison pairing is followed by a unique consequence (outcome). This approach has repeatedly been shown to support quicker learning, higher performance, and more durable retention compared to N‐DOP arrangements (McCormack et al. 2019). However, at present it remains unknown whether these advantages extend to individuals with DLD or other populations with language difficulties.

Further methodological limitations that constrain the ability to draw definitive conclusions include the lack of randomized controlled trials that compare MTS protocols with alternative interventions (e.g., explicit instruction), and the limited number of studies that included follow‐up assessments beyond immediate post‐training probes. Consequently, evidence for long‐term maintenance and real‐world generalization remains scarce. Additionally, given the relatively low baseline language and cognitive skills of participants, training effects were confined to foundational language targets. These limitations underscore the need for further research, a conclusion already highlighted in earlier review(‐like) papers addressing theoretical and empirical issues related to MTS‐based interventions for individuals with language disabilities (Carr and Felce 2000; O'Donnell & Saunders, 2003).

### 4.2 Recommendations for Future Studies

To advance the field and evaluate the potential of MTS‐based interventions for DLD, future research should recruit participants diagnosed with DLD and stratify samples by age, cognitive abilities, and associated linguistic and social skills (Bishop et al. 2017). An ongoing debate concerns the basic cognitive prerequisites for the successful application of MTS‐based interventions, particularly with respect to minimal sensory‐discriminative and language abilities (e.g., Jackson et al. 2006). Nonetheless, it is reasonable to assume that individuals with DLD generally meet these basic requirements, making them a suitable target population for such interventions, although individual differences in severity may affect task feasibility and outcomes.

Factorial study designs that systematically manipulate MTS variables (e.g., OTM versus identity matching, feedback schedules, inclusion of DOP) are needed to identify optimal configurations for distinct language outcomes. Rigorous methodologies—larger samples, randomized controlled trials with clearly defined control groups (e.g., explicit versus implicit training)—will facilitate statistical comparisons and clearer efficacy estimates. Long‐term follow‐up (e.g., one to six months post‐intervention) should assess maintenance of derived relations, while measures of ecological validity (e.g., classroom engagement, conversational use of trained vocabulary or grammar) are essential to demonstrate functional gains.

Perhaps most importantly, extending MTS protocols to more advanced language targets than the basic skills addressed in the reviewed studies could determine whether stimulus equivalence procedures can also scaffold higher‐order linguistic competencies in DLD, including complex syntax and pragmatic discourse. For example, MTS procedures could be designed to strengthen a participant's understanding of complex syntactic rules by using sentence frames with a missing element as samples and written words as comparison options (e.g., The cat that ___ on the wall ran away vs. is sitting, sits, sat). Likewise, pragmatic discourse abilities might be addressed by presenting brief social scenarios (written, pictorial, or video) followed by comparisons representing different discourse moves, of which one is pragmatically appropriate for the context. To address the distinction between implicit and explicit learning processes and their potential involvement in MTS‐based interventions (as outlined in the introduction), future studies could assess individual differences in standard implicit learning tasks measures (e.g., the serial reaction time task) and associated neurobiological markers, and examine how these relate to performance in MTS‐based interventions. Such analyses may elucidate the learning mechanisms involved and help guide more personalized intervention approaches.

## 5 Conclusions

The existing literature demonstrates that MTS‐based training can engender derived stimulus relations and generalization in various neurodevelopmental populations. However, small, methodologically diverse studies limit conclusions regarding the potential utility of MTS protocols for DLD. Rigorous, targeted research, emphasizing larger samples, standardized outcomes, and long‐term functional gains, is essential to determine whether MTS methods can offer an effective, partly implicitly driven complement or alternative to conventional explicit language interventions for individuals with DLD.

## Authors Contributions

**J. Maes**: Conceptualization, Data Curation, Investigation, Methodology, Supervision, Validation, Visualization, Writing—original draft. **A. Scheper**: Validation, Writing—Review & Editing. **D. Hermans**: Validation, Writing—Review & Editing. **C. Vissers**: Validation, Writing—Review & Editing.

## Conflicts of Interest

The authors declare that they have no conflict of interest.

## Ethical approval

Ethical approval was not required for this study as it is a review of published literature

## Data Availability

The dataset documenting the article selection process for the scoping review are openly available via the Open Science Framework under https://osf.io/y7jxh/
